# Suicidality in People With Obsessive-Compulsive Symptoms or Personality Traits

**DOI:** 10.3389/fpsyt.2018.00747

**Published:** 2019-01-14

**Authors:** Rudy Bowen, Hiba Rahman, Lisa Yue Dong, Sara Khalaj, Marilyn Baetz, Evyn Peters, Lloyd Balbuena

**Affiliations:** Department of Psychiatry, University of Saskatchewan, Saskatoon, SK, Canada

**Keywords:** affective dysregulation, mood disorders, anxiety disorders, self-harm, network model, OCD, OCPD

## Abstract

**Objective:** Obsessive-Compulsive Disorder (OCD) and Obsessive-Compulsive Personality Disorder (OCPD) have been reported to be associated with mood instability (MI), depression and suicide-related outcomes. We examined whether obsessive-compulsive symptoms and personality traits as well as obsessional thoughts of death, are associated with suicidal thoughts, non-suicidal self-injury and attempted suicide.

**Methods:** We used data from 7,839 people from the 2000 British Adult Psychiatric Morbidity Survey that elicited symptoms of OCD with a computerized version of the Clinical Interview Schedule—Revised (CIS-R) and traits of OCPD with a self-completed version of the SCID-II. We created a series of logistic regression models, first entering only OCD symptoms and OCPD traits in separate models, to which depression and mood instability (MI) were added. We also examined the relation of obsessional thoughts about death with self-harm in a network analysis model that included the main symptoms of mood instability and depression.

**Results:** OCD symptoms were associated with suicidal thoughts (OR: 1.23, 95% CI: 1.14–1.32), and suicide attempts (OR: 1.13, 95% CI: 1.04–1.24) in the fully-adjusted model. OCPD traits were associated with suicidal thoughts (OR: 1.14, 95% CI: 1.10–1.19), non-suicidal self-injury (OR: 1.14 95% CI: 1.03–1.26), and suicide attempts (OR: 1.09; 1.01–1.17). Depression and MI were both associated with all three suicide-related outcomes. In the network analysis, MI was the most prominent correlate of suicide-related outcomes, being associated with suicidal ideas (partial *r* = 0.15) and non-suicidal self-injury (partial *r* = 0.07).

**Limitation:** This was a cross-sectional study that used a single-item measure for mood instability.

**Conclusions:** Obsessive-compulsive symptoms and personality traits are related to suicide-related outcomes independently of depressive symptoms and mood instability. This relationship is not accounted for by obsessional thoughts of death alone.

## Introduction

The World Health Organization estimates that more than 800,000 deaths per year occur because of suicide (1). Deficiencies in the delivery of health care have been identified as possible causes, but even in developed countries, the suicide rate has not decreased in the past 5 years, on the contrary, the rate appears to be increasing again (2). Almost all psychiatric conditions are associated with suicide including anxiety and mood problems, schizophrenia, eating disorders, substance abuse, and personality disorders (3–7) but depression is the most common psychiatric condition that leads to suicide (8–10). It is now apparent that increasing the availability of psychological and pharmacological treatments has not decreased the prevalence of depression (11).

Mood instability (MI) (12, 13) and recurring intrusive distressing thoughts (repetitive negative thinking) are two frequent trans-diagnostic co-occurring conditions with depression (14–16). MI refers to rapid and intense variability in mood that occurs over relatively short periods of time (often within hours) (17–19). It is reported to be an antecedent of suicidal thoughts and depression (20–24). In the psychological literature a concept similar to MI is referred to as emotional dysregulation, although we believe that MI refers to a more specific, measurable entity (25). A high rate of MI has been shown in common mental disorders/neurotic conditions and specifically in participants with obsessive-compulsive disorder (OCD) and obsessive-compulsive personality disorder (OCPD) (19, 26).

Repetitive negative thinking includes rumination about the current psychiatric condition and unpleasant perseverative thinking about past and future events (14). Recurring thoughts of death and suicide (27) and aggression (28, 29) are common features of OCD. The most prevalent obsession was the fear of harming oneself in the DSM-IV field trials of 431 patients diagnosed with OCD (30, 31). Several other studies have indicated an association between OCD and suicidal thoughts (32–36) which led the authors of a comprehensive review to suggest theory- driven studies to investigate suicidality in OCD (37). Suicidal ideation or attempts by people with OCD may be partly driven by a pathological belief in one's ability to control negative outcomes causing distress and MI (38). When OCD is comorbid with alexithymia, the delusion of control and its accompanying guilt may be exaggerated making suicide appealing (38).

Personality disorders may be in a continuum both with mental state disorders (i.e., Axis I) disorders and with themselves (39, 40). There are many similarities between OCD and OCPD that justify studying them together. OCD, OCDP and major depression frequently occur together (40). Common characteristics include heritability, manifestations, clinical course, and response to specific serotonin reuptake inhibitor antidepressants (41). Preoccupation with details, perfectionism and rigidity are most characteristic of OCPD (41) and OCPD traits are directly related to the severity of OCD (42). In light of the recommendation by the Research Domain Criteria (RDoC) framework for psychiatric entities to be informed by the basic sciences (43), we treated OCD and OCPD as falling within the same spectrum as suggested by others (44, 45).

There are theoretical distinctions between suicidal thoughts, non-suicidal self -injury (NSSI), and suicide attempts, but when cohorts of people with NSSI are studied, the same individuals tend to engage in both NSSI and suicide attempts (46–48). Because of this, we addressed suicidal thoughts, NSSI and suicide attempts as related outcomes. The interpersonal theory of suicide provides a link between thoughts and acts in the presence of feelings of burdensomeness, lack of belongingness and loss of the fear of death (10, 49). Repetitive negative thinking about the state of being depressed and death, as occurs in OCD or OCDP, might also desensitize the person to the fear of death (29, 31, 50).

Based on the literature and reasoning above we proposed the following three hypotheses within a group of individuals from the general population with OCD and OCPD: (1) That OCD symptoms and OCPD traits would predict suicide-related thoughts, NSSI, and suicide attempts; (2) that in addition, MI and depression would predict all three suicide related outcomes; (3) that obsessive thoughts dwelling on death would make suicide attempts more likely.

## Methods

### Materials

The British Adult Psychiatric Morbidity Survey (APMS) of 2,000 was the second of a series of surveys intended to monitor the prevalence, disability and treatment of psychiatric illness in England, Scotland, and Wales. An overview is provided here but details are contained in earlier reports (51, 52).

The sample consisted of 7,839 people, aged 16–74 years, living in private households, selected randomly from postal codes of small user postcode address files (delivery points receiving <50 items of mail each day), stratified by region and socio-economic group. One eligible person was selected randomly from each household using the Kish Grid method (53). A small number of individuals living in institutions were not included. At the initial interview 69.5% agreed to participate and 90% of these completed the full interview (54).

Since we used publicly available, anonymous data, no ethical review was required for this study, but ethical approval was obtained for the original study from the London Multi-Center Research Ethics Committee and from each of the 149 local research ethics committees.

### Measures

The main instrument used to establish symptoms of OCD was the CIS-R, using computer-assisted interviewing techniques. This is a fully structured interview, administered by trained non-clinical interviewers from the Office of National Statistics (55). Training was provided by senior trainers from the Office of National Statistics and a psychiatrist (56). Interviews were conducted between March and July in two stages. The material for this study was all from the first stage of the interview. The CIS-R uses initial filter questions to establish the presence of key symptoms in the past month, leading to more detailed assessment of individual symptoms that occurred over the past week. This interview took about 90 min (54).

Our outcomes of interest were suicidal thoughts, non-suicidal self-injury and suicide attempts. We selected these outcomes because they can be thought of as crossing over from thought to action, not remaining as an intention. NSSI may be one way of coping with negative emotions but also serve as habituation to the possibility of death (57, 58). The section on deliberate self-harm asked about lifetime experience with these thoughts and actions. Eight questions were asked about obsessions and compulsions (34, 59). Obsessions related to the respondent's experience of repetitive thoughts or ideas over the past week, while compulsions related to doing things repeatedly, also over the past week.

After the initial interview, participants were given the self-report version of the SCID-II interview to be completed and mailed in (59). We used the eight symptoms given in DSM-IV and DSM-5 to elicit traits of OCPD (60, 61). MI was assessed by criterion 6 from the Borderline Personality Disorder section of this interview, “affective instability due to a marked reactivity of mood…” (59). OCPD traits and MI referred to lifetime experience of these thoughts and events.

The participants who reported having repetitive thoughts or ideas were also asked about the content of these thoughts. These were open-ended responses that were coded independently by two of the co-authors (SK and YD) as “1” if death-related and “0” otherwise. The kappa statistic for the ratings was 0.77, *p* < 0.001. According to Landis and Koch kappa values between 0.61 and 0.80 are interpreted as being in substantial agreement (62).

### Statistical Analysis

To answer questions 1 and 2, we developed survey-weighted logistic regression models with respect to our three outcome variables: suicidal thoughts, self-harm without intent to kill, and suicide attempts. We created regression models in a stepwise fashion, first examining the gross effect of OCD (or OCPD) and then examining the net effect by adjusting for mood disorders with reported associations for suicide-related outcomes (63–65). First, the count of OCD symptoms (Table 1. Model 1) or OCPD traits (Table 2. Model 1) was entered adjusting only for sex and age. Then the count of depression symptoms (0 to 7, omitting the suicidal thoughts item) was entered (Table 1. Model 2 and Table 2. Model 2). Finally, MI was entered (Table 1. Model 3 and Table 2. Model 3) in a fully adjusted model. Collinearity among predictor variables was tested using the variance inflation factor statistic (VIF), with VIF > 10 indicative of collinearity. Regression modeling was implemented in Stata.

**Table 1:** Weighted logistic regression models: OCD and suicide-related outcomes (Odds Ratios, 95% CI, *p*-value).

	**Suicidal thoughts**	**Self-harm without intent to kill**	**Suicide attempt**
**Model 1:**			
OCD symptom count (range: 0–8)	1.53 (1.44–1.64), *p* < 0.001	1.40 (1.28–1.54), *p* < 0.001	1.47 (1.38–1.57), *p* < 0.001
**Model 2:**			
OCD symptom count (range: 0–8)	1.29 (1.20–1.38), *p* < 0.001	1.16 (1.03–1.30), *p* = 0.01	1.19 (1.10–1.28), *p* < 0.001
Depression symptoms (0–7)	1.36 (1.31–1.41), *p* < 0.001	1.37 (1.27–1.47), *p* < 0.001	1.41 (1.34–1.49), *p* < 0.001
**Model 3:**			
OCD symptom count (range: 0–8)	1.23 (1.14–1.32), *p* < 0.001	1.10 (0.98–1.25), *p* = 0.11	1.14 (1.04–1.24), *p* < 0.01
Depression symptoms (0–7)	1.27 (1.23–1.32), *p* < 0.001	1.25 (1.15–1.36), *p* < 0.001	1.31 (1.23–1.39), *p* < 0.001
Mood instability	2.84 (2.37–3.41), *p* < 0.001	3.30 (2.13–5.10), *p* < 0.001	2.79 (2.04–3.85), *p* < 0.001

**Table 2:** Weighted logistic regression models: OCPD traits and suicide-related outcomes (Odds Ratios, 95% CI, *p*-value).

	**Suicidal thoughts**	**Self-harm without intent to kill**	**Suicide attempt**
**Model 1:**			
OCPD traits total (range: 0–8)	1.28 (1.23–1.33), *p* < 0.001	1.33 (1.22–1.46), *p* < 0.001	1.27 (1.19–1.35), *p* < 0.001
**Model 2:**			
OCPD traits total (range: 0–8)	1.19 (1.14–1.24), *p* < 0.001	1.20 (1.09–1.33), *p* < 0.001	1.14 (1.06–1.22), *p* < 0.001
Depression symptoms (0–7)	1.38 (1.33–1.43), *p* < 0.001	1.36 (1.28–1.46), *p* < 0.001	1.44 (1.37–1.51), *p* < 0.001
**Model 3:**			
OCPD traits total (range: 0–8)	1.14 (1.10–1.19), *p* < 0.001	1.14 (1.03–1.26), *p* = 0.01	1.09 (1.01–1.17), *p* = 0.02
Depression symptoms (0–7)	1.30 (1.25–1.35), *p* < 0.001	1.26 (1.17–1.36), *p* < 0.001	1.33 (1.26–1.41), *p* < 0.001
Mood instability	2.75 (2.30–3.28), *p* < 0.001	3.03 (1.97–4.67), *p* < 0.001	2.76 (2.03–3.76), *p* < 0.001

To answer question 3, we created a network model based on partial correlations of selected symptoms with the suicide-related outcomes. We selected the core symptoms of depression in the DSM-5 model of depression which are lack of interest and feeling sad miserable or depressed. We also selected thoughts of taking one's life, non-suicidal self-injury, and suicide attempts which are the range of suicidal thoughts and actions that we studied plus items representing mood instability, worrying too much, obsessing about death and compulsivity. We reasoned that a network model would show a direct connection between symptoms (represented as nodes) if a partial correlation between the symptoms was significant. Alternatively, the lack of a direct link between symptoms would indicate that they are only related indirectly. We were particularly interested in whether obsessional thoughts about death would be related directly to the suicidal outcomes. Network modeling was carried out in R using the qgraph package (66). In particular, our network model used partial correlations of mood symptoms and suicidality using an adaptive lasso penalty.

## Results

### OCD and Suicide-Related Outcomes

OCD symptoms were associated with all three outcomes adjusting for age, gender, and depression symptoms (Table 1. Models 1 and 2). When MI was entered, OCD symptoms were related to suicidal thoughts and suicide attempts but not to self-harm without intent to kill. Depression symptoms and MI were related to all three suicide-related outcomes (Table 1. Model 3). The fully adjusted OCD model did not show collinearity among predictor variables, VIF = 1.08.

### OCPD Traits and Suicide-Related Outcomes

OCPD traits were associated with all three suicide-related outcomes after adjusting for age, gender, depression symptoms, and mood instability (Table 2. Models 1 to 3). Depression and mood instability were also significantly related to suicide-related outcomes. The fully adjusted OCPD traits model did not show collinearity among predictor variables, VIF = 1.07.

### Network Analysis of Mood Symptoms

**Figure 1:**
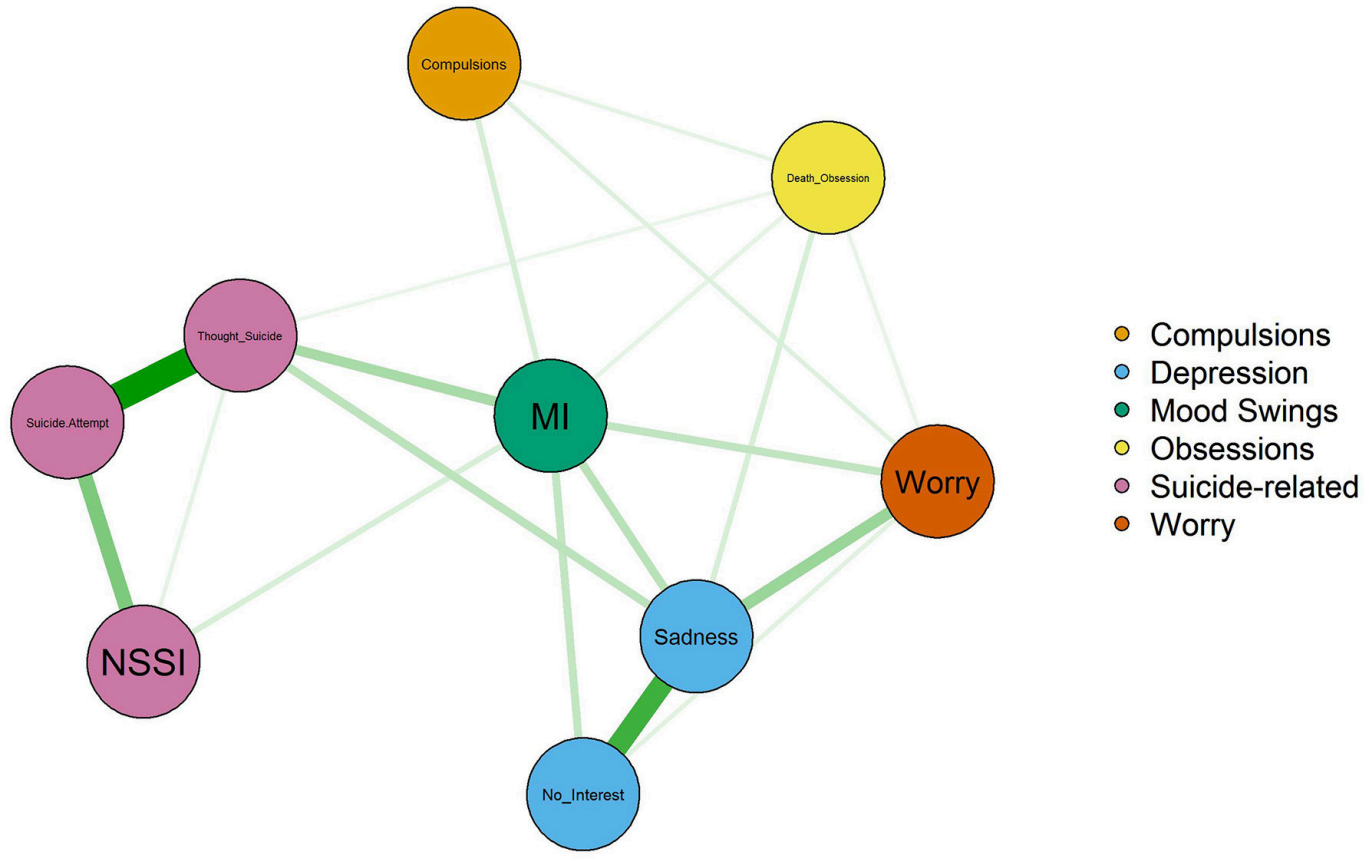
Network model of suicidal outcomes and mood symptoms.

Please refer to Table 3 and Figure 1. Obsessional thoughts about death were only very weakly related with thinking about taking one's own life (partial *r* = 0.04) after accounting for mood swings (partial *r* = 0.15) and depression (partial *r* = 0.12). Suicide attempts were related to thoughts of taking one's life (partial *r* = 0.46) and self-harm without intent to kill oneself (partial *r* = 0.23). MI was also related to NSSI (partial *r* = 0.07) and thoughts of taking one's life (partial *r* = 0.04).

**Table 3:** Partial correlations of mood symptoms.

	**1**	**2**	**3**	**4**	**5**	**6**	**7**	**8**	**9**
1. Worry too much	1	0.18	0.05	0.06	0.04	0.11	0	0	0
2. Sad, miserable, depressed (in the past week)		1	0.35	0.00	0.07	0.12	0.12	0	0
3. Lack of interest			1	0	0	0.11	0.00	0.00	0.02
4. Does things repeatedly 4 days/wk. Compulsions				1	0.05	0.07	0.00	0.00	0.00
5. Intrusive thoughts of death (obsess-death)					1	0.04	0.04	0	0
6. Mood instability (MI)						1	0.15	0.07	0.00
7. Thought of taking life (suicidal thoughts)							1	0.04	0.46
8. Harmed self with no intent to kill (NSSI)								1	0.23
9. Attempted to take life (suicide attempt)									1

## Discussion

The main finding is that OCD symptom scores and OCPD trait scores predict suicidal thoughts, NSSI (with the exception of OCD symptoms and NSSI) and suicide attempts, with depression and MI controlled. A previous report from the Adult Psychiatric Morbidity Survey (34) and several reviews of the literature have confirmed a link between OCD and suicidal behavior (37, 67, 68). The literature indicates that the strength of the association between OCD and suicidality increases with comorbidity with anxiety and depression and past history of suicidality (37).

Several possible theories are proposed to explain the association between OCD and OCDP and suicidal thoughts (37). Repetitive negative thinking about harm to the body might increase tolerance of distress at thoughts of pain (2, 15, 49). It is relevant that thoughts of harming oneself or others (along with sex and unacceptable urges) were the most common obsessions among the predominantly obsessional group (30% of the total group) in the DSM-IV field trials of 431 patients with OCD (30, 31). Added psychological burdens accompanying OCD and OCDP are interference with personal relationships and inability to work (37).

The second important finding is that mood instability (MI) is the main predictor of suicidal thoughts and behaviors, even when depression is controlled. This is consistent with previous work from the APMS 2007 that showed that both MI and OCD show an association with suicidality (19). It has been reported that instability in repetitive negative thinking contributes more to non-suicidal self-injury rather than stable patterns of thinking (30, 69). We have previously shown that MI along with negative affect (depression, anxiety and irritability) is the main predictor of suicidal thoughts, using national data from the Netherlands (20). Depression (meaning a more pervasive low mood), can be a consequence of the frequent, unpredictable, sudden drops in mood that constitute MI (23). It is possible that personal and social problems associated with frequent and unpredictable shifts in mood lead to decreased sense of belongingness and increased burdensomeness (49).

The third finding is a negative result from the network analysis of mood disorder symptoms. We did not find support for the hypothesis that obsessions about death make suicide attempts more likely. (Please refer to Table 3 and Figure 1). In Table 3 and Figure 1, note that obsessing about death is weakly linked to suicidal thoughts, but not to self-harm or suicide attempts. Consistent with the findings from the regression analysis, we found that MI serves as a bridge from various mood disorder symptoms to the suicide spectrum. The findings suggest that it is the total psychological burden of OCD or OCDP plus MI plus pervasive depression that contributes to self-harm, rather than only thoughts or actions of harming the self.

In Table 3 and Figure 1, MI is also linked to worry. Intrusive thoughts of death as might occur in OCD are only weakly connected to thoughts of taking one's life and suicide attempts. Examples of thoughts of death taken from the data are “hoping I go to sleep and don't wake up” “wishing it was all over” “I'm obsessed by death….” This finding is consistent with the clinical guideline that asking about suicidal thoughts does not appear to increase the risk for suicide (70–72).

The main limitations of this study were the use of a single question for assessing MI and the retrospective study design. Generally, single-item measures are unfavorable because it is not possible for internal consistency (a form of reliability) to be assessed. However, it is possible in some cases to estimate its reliability relative to that of a multi-item scale, by using the correction for attenuation formula (73). Unfortunately, it was not possible to do so in the present work because the APMS 2000 did not have a gold standard measure of mood instability (i.e., the unstable moods factor derived from Eysenck neuroticism (12) or did not have sufficient cases (i.e., bipolar disorder). With regard to recall bias, the assessment of MI may be reliable and valid if specific questions are asked (74). It is also similar to questions used in clinical situations and personality inventories such as the short form of the Eysenck Personality Inventory (75).

Our study has the advantage of being based on a large epidemiologic survey and validated questionnaires for every trait would not have been feasible. The question used to assess MI appeared readily comprehensible to participants and had good face validity; only 25 people in 2000 and 48 people in 2007 declined to answer it. This question has been used in published studies to substantiate hypotheses related to depression and PTSD (76), psychosis (77) and bipolar disorder (78) suggesting construct validity. It would be important in future work to establish its reliability compared with a gold standard. The question of validity of psychiatric interviews conducted by lay interviewers compared with those done by clinicians is an ongoing concern (56). With OCD, one problem in interviews is the emphasis on compulsions which are more readily observed rather than obsessions which are harder to access. The APMS data placed as much emphasis on recurring thoughts (obsessions) as on compulsions. The recurring thoughts were also recorded verbatim (79). With regard to self-completed data in the personality assessment or the lay interviewer completed CIS-R (56), the understanding of some concepts of mental health by members of the public might be limited (56). This study was also cross-sectional and conclusions should be viewed in relation to those of related longitudinal studies (80, 81).

It is possible that the gene or genes involved in the obsessive spectrum will be discovered which will suggest more specific therapies. In the meanwhile, alexithymia has been suggested as a clinically useful concept in predicting suicide in patients with the obsessive spectrum (38). Alexithymia overlaps with depression which is among the varying moods of MI (82), but reasons for the relationship including brain changes still require clarification (83). The results suggest that repetitive negative thinking should be better recognized as a trans-diagnostic problem which would include thoughts of recurring worthlessness or excessive guilt (criterion 7 of major depression), excessive worry (the main criterion for generalized anxiety disorder), intrusive re-experiencing (Criterion B for post-traumatic stress disorder) (61), all of which are associated with an increased suicide rate. Increased recognition and more attention to MI, which is also a trans-diagnostic symptom could contribute to suicide prevention. Perhaps the rate of suicide could be reduced if obsessions were recognized as the main feature of the OCD spectrum instead of compulsive acts (84). A dimensional conceptualization of mental disorders would allow for symptoms that are central to any given condition to also be present in other conditions (48, 85).

## Author Contributions

RB conceptualized the study, wrote the initial and final drafts. HR performed a systematic literature review and provided the theoretical basis for the study. SK provided clinical insights that contributed to conceptualize the study. LD performed the statistical analysis and wrote the methods and results of the initial draft. EP helped interpret the results and critically revised the manuscript. MB critically reviewed the results and contributed to the discussion. LB assisted in the statistical analysis and re-wrote the initial draft. All authors reviewed and approved the submission.

### Conflict of Interest Statement

The authors declare that the research was conducted in the absence of any commercial or financial relationships that could be construed as a potential conflict of interest.
